# Lean leadership attributes: a systematic review of the literature

**DOI:** 10.1108/JHOM-12-2016-0245

**Published:** 2017-10-09

**Authors:** Kjeld Harald Aij, Maurits Teunissen

**Affiliations:** 1 Department of Anesthesiology and Operative Care, VU University Medical Center, Amsterdam, The Netherlands; 2 VU University Medical Center, Amsterdam, The Netherlands

**Keywords:** Leadership, Management, Healthcare, Management attitudes

## Abstract

**Purpose:**

Emphasis on quality and reducing costs has led many health-care organizations to reconfigure their management, process, and quality control infrastructures. Many are lean, a management philosophy with roots in manufacturing industries that emphasizes elimination of waste. Successful lean implementation requires systemic change and strong leadership. Despite the importance of leadership to successful lean implementation, few researchers have probed the question of ideal leadership attributes to achieve lean thinking in health care. The purpose of this paper is to provide insight into applicable attributes for lean leaders in health care.

**Design/methodology/approach:**

The authors systematically reviewed the literature on principles of leadership and, using Dombrowski and Mielke’s (2013) conceptual model of lean leadership, developed a parallel theoretical model for lean leadership in health care.

**Findings:**

This work contributes to the development of a new framework for describing leadership attributes within lean management of health care.

**Originality/value:**

The summary of attributes can provide a model for health-care leaders to apply lean in their organizations.

## Introduction

Rising health-care costs have been attributed to factors including aging populations, increasing prevalence of chronic diseases such as obesity, diabetes, and cancer, expensive technologies and pharmaceuticals, and increasing complexity. Simultaneously, emphasis on quality has led many health-care organizations to reconfigure their management, process, and quality control infrastructures. One popular model used for these reconfigurations is lean, a management philosophy and process methodology derived from the Toyota Production System (TPS) (Womack *et al.*, 1990).

Lean was developed to increase operational efficiency by addressing quality, speed, and cost (Holweg, 2007). Lean focuses on elimination of waste (*muda*), unevenness (*mura*), and overburden (*muri*) (Womack, 2006). Lean’s origins are found in the process improvement strategies designed by Henry Ford (Womack and Jones, 1996), which were further refined by engineers at Toyota Motor Company between 1949 and 1975, resulting in TPS. Researchers from the Massachusetts Institute of Technology (MIT) identified the TPS as best practice and coined the term “Lean” to describe this way of doing things (Krafcik, 1988; Womack *et al.*, 1990).

While widely used in manufacturing industries since the 1980s, the application of lean to health care was not proposed until 1996 by Womack and Jones. Since then, lean has been widely – if often partially – implemented in hospitals and health systems and has become the approach most frequently reported in the health-care management literature, accounting for more than half of all references (Mazzocato *et al.*, 2014). This growing body of research shows that successful lean implementation requires system-wide change and that effective leadership is necessary to catalyze and sustain that change (Aij, Aernoudts and Joosten, 2015; Al-Balushi *et al.*, 2014; Dombrowski and Mielke, 2014; Ghosh and Sobek, 2015).

In a lean organization, leadership ensures all employees are fully engaged and involved in daily improvement activities (Dombrowski and Mielke, 2013). Conversely, lean initiatives often fail when leaders are not able to grasp and convey the need for systemic change (Al-Balushi *et al.*, 2014; Waring and Bishop, 2010) or to manage by process, rather than outcome (Toussaint, 2015). In health-care settings, value is defined from the perspective of the patient. The shift to lean thinking requires development of a culture in which employees are empowered and encouraged to make improvements (Drotz and Poksinska, 2014). The application of lean to health care, as many have argued, must be modified because of the high variability of the “product” – health for unique individuals with unique constellations of symptoms – in comparison to the consistent sameness of manufacturing products (Renaud, 2014).

Most existing lean health-care literature focuses on the implementation of lean in an organization; leadership is acknowledged as important, but specific attributes and behaviors of successful lean leaders in health care have been poorly defined. The literature of leadership behavior in lean organizations is missing (Mann, 2014; Dombrowski and Mielke, 2013).

### Theoretical background

Dombrowski and Mielke (2013) proposed a conceptual model for an integrated lean leadership system. Their conceptual model, which is not industry specific, includes five core principles of lean leadership: improvement culture, self-development, qualification, *gemba*, and *hoshin kanri*. The five components all contribute to the core concept of the team as the central active unit in a lean environment. Teams that include front-line workers are central to employee engagement and process improvement. Each of the five principles is described in the following paragraphs.

Improvement culture encompasses all attitudes and behaviors that create an ongoing striving for perfection. While leaders seek to prevent failure, when failure does occur, it is seen as an opportunity to improve. Failures are investigated for their root causes, which are addressed to ensure that the failure does not occur again. All people in the organization – employees, management, board – are part of an improvement culture. Front-line workers enact the processes, while leaders coordinate the problem solving and process management of the team.

The need for self-development is based on awareness that the transition to lean leadership demands new leadership skills, some of which are innate, and some of which must be learned. Lean leaders must behave as role models and use the necessary leadership skills. Both leaders and workers are mentored by a *sensei*, who provides objective feedback and guidance. Leaders must also learn to use specific lean tools, such as plan-do-check-act cycles.

Qualification of employees involves fostering employee involvement and learning, often in apprentice-style learning. Qualified employees are better able to participate in continuous improvement, problem solving, and other lean activities. This process should be guided by a coach to help establish daily routines and to develop a sustainable continuous improvement environment.

*Gemba*, the fourth principle of Dombrowski & Mielke’s lean leadership model, requires that leaders go to the place where value is created. The Japanese word *gemba* literally means “real place” and, in lean terms, refers to the environment where value-adding processes occur (Liebengood *et al.*, 2013). As a leadership principle, *gemba* refers to the necessity for lean leaders to understand what happens on the front lines, and to understand the problems and processes that their employees deal with. Regular *gemba* walks also show the leader’s appreciation for the work of employees in creating that value. Lean leaders follow five “golden *gemba* rules” when a problem occurs: go to *gemba*; check; take temporary countermeasures; find the root cause; and standardize. Facility with these five steps is a core leadership skill.

*Hoshin kanri*, sometimes called “target management” or “policy deployment,” is a method of aligning goals with customer focus on all levels. *Hoshin kanri* uses a system approach to improvement, combining all teams to align with the same strategic goal. Although each team has a different short-term goal, *hoshin kanri* provides the overarching direction for teams in combination to achieve long-term goals. Lean leaders develop long-term strategies and goals and coordinate the work of teams.

**Figure 1:**
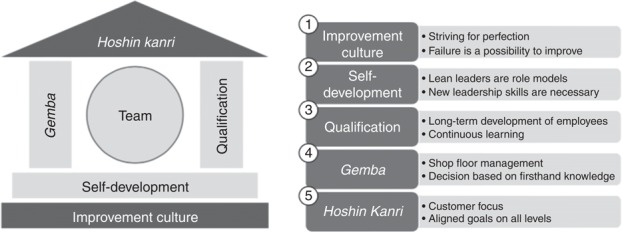
Dombrowski and Mielke’s lean leadership model

Dombrowski and Mielke’s leadership model is depicted in Figure 1.

### Research objective

While Dombrowski and Mielke provide an overview of lean leadership actions and behaviors, they offer little insight into the attributes and characteristics of effective lean leaders. In addition, their model is not specific to health care. Few researchers have probed the question of ideal leadership attributes to achieve lean thinking in health care. We seek to fill that gap by systematically reviewing the literature on principles of leadership and, using Dombrowski and Mielke’s (2013) conceptual model of Lean leadership, developing a parallel theoretical model for lean leadership in health care.

## Methods

We conducted a systematic narrative review of published articles about lean leadership, other leadership models, and health care to assess attributes of lean leaders and their contribution to lean work environments. We systematically searched relevant terms using the MEDLINE (accessed through PubMed), EMBASE, and Emerald databases. Search syntax was based on the variables identified in the research question: attributes, lean leadership, leadership, and health care. Initial inclusion criteria were English-language articles describing an empirical study or comprising a theoretical secondary review published in peer-reviewed journals. Dates were restricted to 2000-2016, as lean implementation in health care began during the early 2000s. Articles that did not address leadership or health care were excluded, as were papers that did not discuss attributes of leaders. Table I provides an overview of inclusion and exclusion criteria.

Keywords were differentiated to find the most relevant literature. For the initial search, the syntax was “lean leadership” or “leadership” and “attributes,” “traits,” “characteristics,” “competences,” and “health care.” These elements were combined into the following final search syntax: (lean [All Fields] AND (“leadership” [MeSH Terms] OR “leadership”[All Fields])) AND (traits [All Fields] OR characteristics [All Fields] OR competences[All Fields] OR attributes [All Fields]) AND lean [All Fields] AND (“health” [MeSH Terms] OR “health” [All Fields]) AND care [All Fields] AND (“delivery of health care” [MeSH Terms] OR (“delivery” [All Fields] AND “health” [All Fields] AND “care” [All Fields]) OR “delivery of health care” [All Fields] OR “healthcare” [All Fields]).

**Figure 2:**
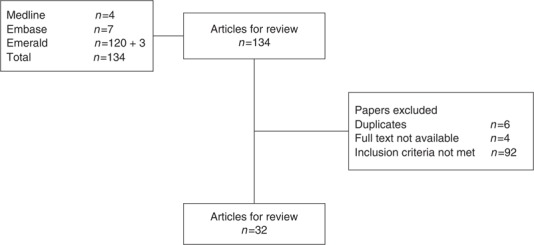
Article selection procedure

A total of 131 potentially relevant references were identified; 4 articles were identified via MEDLINE, 7 via EMBASE, and 120 via Emerald. The relevance of each article was determined by reading the abstract, results, and conclusion. Initial screening identified 6 duplicate articles, 4 articles for which full text was not available, and 91 articles that did not meet inclusion criteria. Also, three additional articles were identified through a search for a leading researcher in the field; one article was not peer reviewed and was thus excluded. The remaining 32 articles were read in full and coded to assess for the five principles of lean leadership described by Dombrowski and Mielke (2013). “Attributes” were matched to “improvement culture,” “self-development,” “qualification,” “*gemba*,” and “*hoshin kanri* (Figure 2).”

## Results

A total of 32 articles met the inclusion criteria and were included in the review (see www.researchgate.net/profile/Kjeld_Aij). These included 11 papers pertaining to lean leadership and 21 papers that discussed other leadership styles. Study designs varied widely and included two systematic and four informal literature reviews; eight analyses of case studies, two surveys, four interviews; four viewpoint articles; four theoretical analyses; and seven concept papers. Several articles combined methods, such as literature reviews with interviews or case studies. Of the 32 articles, 30 relied primarily on qualitative data.

Results were categorized based on the five principles of lean leadership identified by Dombrowski and Mielke (2013), and associated attributes in health care were described as follows (Tables II-VI):

1. Improvement culture: task identity, feedback, autonomy, belief in improvement, and honesty were the core attributes of leaders who successfully implemented an improvement culture. These attributes emphasize the need for leaders to recognize the importance of involving employees in lean initiatives, to embrace improvement models themselves, and to honestly recognize failures and see them as opportunities for improvement. Employees often recognize errors in the work processes but are not able to fix them on their own. The lean leader should support employees to enhance improvement at all levels of the hierarchy of the organization.
2. Self-development: successful lean leaders are open to developing their own competencies and skills. Some attributes required to be a lean leader depend on the leader’s personality, and other attributes have to be learned and developed. Self-development was connected to the attributes show interest, facilitate resources, emotional intelligence, visualize greatness, be aware of one’s status, and have the skills to motivate, inspire, stimulate, and facilitate (Al-Balushi *et al.*, 2014; Hopkins *et al.*, 2011; Kent, 2006; Millward and Bryan, 2005). The literature did not categorize the attributes; however, attributes that support self-development create the mechanism for leaders to become role models.
3. Qualification: qualification attributes found in the literature were: empowerment, trust, communication, clarify, governance and consistency, and fit for purpose (Albrecht and Andreetta, 2011; Antony *et al.*, 2007; Kent, 2006; Marinelli-Poole *et al.*, 2011; Steed, 2012; Willis *et al.*, 2016). These attributes enable leaders to support employees’ development, both individually and collectively. Development of employees usually occurs through training, but in lean thinking, development at the place where the work happens is also critically important (Dombrowski and Mielke, 2013).
4. *Gemba*: the lean leader retains the *gemba* as the place of learning and action (Dombrowski and Mielke, 2014) and performs regular *gemba* walks publicly with care, recognition, engagement, a focus on leader-employee relationships, communication, and fairness (Aij, Visse and Widdershoven, 2015; Braithwaite *et al.*, 2007; Glavin and Chilingerian, 2010; Kent, 2006; Steed, 2012). The leader gains trust of employees by demonstrating engagement and showing himself to be honest, benevolent, and express well-intentioned behavior (Braithwaite *et al.*, 2007). In this way, trust and confidence are built in the leader-employee relationship. Both verbal and non-verbal communication help to build trust. Although *gemba* refers to the place where the work happens, the purpose of the attributes is not to find the root cause of problems. Increased interaction with employees, however, will lead to better identification of mistakes and errors in processes.
5. *Hoshin Kanri*: the lean leader is ultimately responsible for implementing the principle of *hoshin kanri* in the organization. Leadership attributes associated with *hoshin kanri* were define and provide value; demonstrate principles of lean; communication; and role adaptation (Al-Balushi *et al.*, 2014; Angood and Shannon, 2014; Drotz and Poksinska, 2014; Marinelli-Poole *et al.*, 2011; Pronovost and Marsteller, 2014; Steed, 2012). These attributes help the leader to focus on continuous improvement processes and strategic alignment of activities, processes, and goals, maintain a customer focus, and act within the hierarchical structure of the health-care organization.

## Discussion

The purpose of this systematic literature review was to provide insight into applicable attributes for lean leaders in health care. Using the leadership model developed by Dombrowski and Mielke (2013), we identified corresponding attributes of leadership described in lean and other health-care leadership literature. Applicable attributes for lean leaders in health care have been identified through the application of five principles of lean leadership: improvement culture, self-development, qualification, *gemba*, and *hoshin kanri*. In addition, we found significant overlap between attributes, many of which contribute to the realization of more than one principle.

### Principles and attributes

#### Improvement culture

All articles in this review directly or indirectly addressed the importance of the leader’s ability to foster and maintain an improvement culture (Dombrowski and Mielke, 2013) across the health-care organization. Toussaint and Berry (2013) define lean itself as “an attitude of continuous improvement.” (p. 75). Our review identified four elements that lean leaders should use to improve the culture: task identity, feedback, autonomy, belief in improvement, and honesty (Caldwell, 2014; Crump, 2008; Dannapel *et al.*, 2014; Drotz and Poksinska, 2014; Steed, 2012; Toussaint and Berry, 2013). Task identity is the extent to which an employee completes a whole unit of work and perceives the individual contribution to the entire product (Drotz and Poksinska, 2014). In the lean environment, feedback on job performance is offered through visual control and daily management. Lean leaders must provide the infrastructure for feedback to occur in the workplace, allocating time, resources, and creating supportive structures. Feedback should be complemented by autonomy, which provides team members with a sense of agency and respect. Drotz and Poksinska (2014) differentiate between responsible autonomy – “the extent to which an employee has responsibility and decision-making authority” – and choice autonomy – “the extent to which an employee has freedom concerning work procedures and timing” (p. 180). Leaders can foster autonomy in health-care workers by developing and training self-managed teams, decentralizing authority, sharing power, and empowering teams to participate in decision making (Caldwell, 2014; Crump, 2008; Drotz and Poksinska, 2014). By supporting autonomy, leaders help employees feel connected to the team and make an effort to exceed and not accept complacency (Berg and Black, 2014; Caldwell, 2014; Steed, 2012). To achieve a culture of continuous improvement, senior management must allow employees who are closest to the problems to be solved to take on the role of “master problem solver” (Toussaint and Berry, 2013, p. 75).

Effective leaders believe in improvement (Crump, 2008; Kent, 2006; Mazzocato *et al.*, 2014; Micallef and Straw, 2014; Millward and Bryan, 2005). Leaders should be able to articulate the results they wish to see from improvement activities (Kent, 2006). Physicians can be especially effective in this capacity, as their clinical knowledge can create evidence-based arguments for improvement activities (Angood and Shannon, 2014). Organizational culture is often seen as a building block (Berg and Black, 2014), and the lean leader must ensure that culture is devoted to quality.

Honesty was identified as an essential attribute in both lean and other leadership models (Caldwell, 2014; Steed, 2012). In the lean environment, failure is seen as a possibility to improve, and honesty permits leaders to admit and learn from errors (Aij, Aernoudts and Joosten, 2015; Al-Balushi *et al.*, 2014). Honest leaders help to build a culture in which all workers embrace the approach to identifying errors in processes without blaming individuals. In the lean culture, “no problem” is a problem (Toussaint and Berry, 2013).

#### Self-development

Out of 32 papers in this review, 30 noted the need for self-development of leaders, whether in the form of formal training, hands-on experience, or coaching. As a principle of lean leadership, self-development rests on the premise that lean leaders must present themselves as role models. Self-development was connected to the following attributes: show interest, facilitate resources, emotional intelligence, visualize greatness, be aware of status, and develop skills (Marinelli-Poole *et al.*, 2011; Sloan *et al.*, 2015; Toussaint and Berry, 2013).

Al-Balushi *et al.* (2014) found that effective lean leaders show interest in employees and their work; these leaders also ensure that resources are available for employees to establish lean initiatives. Hopkins *et al.* (2011) propose that leadership development programs that integrate emotional intelligence and coaching are essential to developing the human elements of leadership. Like Al-Balushi’s team, they note the importance of mobilizing resources – for instance, allowing physicians to participate in a leadership development program while on the clock.

Leaders who visualize greatness are also more successful in achieving results (Kent, 2006). In his description of leadership factors necessary for self-development, Kent (2006) focuses on visualizing greatness and managing one’s self. Successful leaders visualize a positive future, like the athlete who visualizes himself crossing a finish line. They create and communicate a shared vision of the future, which “motivates, focuses, and creates a sense of value and significance” (p. 54). Managing one’s self is the capacity to maintain awareness of one’s own behavior, thinking, and feelings, and to consciously choose how one presents oneself to others.

Lean leadership literature specifically mentioned the use of a *sensei*, or mentor, as a coach, while diverse coaching models were described in other literature reviewed. Hopkins *et al.* (2011) note that coaches give feedback on the development of new behaviors, increasing emerging leaders’ self-awareness. Extended dialog between the leader and the coach may enhance emotional intelligence.

Leaders need the skills to motivate, inspire, stimulate, and facilitate others’ development (Millward and Bryan, 2005), and they also need to be aware of the need to acquire those skills. These skills are essential to the lean leader’s core work of empowering team members, and when leaders apply these skills, staff is more likely to make the changes necessary for lean implementation (Al-Balushi *et al.*, 2014).

#### Qualification

The principle of qualification in lean leadership is defined as the long-term development of employees and their continuous learning (Al-Balushi *et al.*, 2014; Dombrowski and Mielke, 2013). This review showed that following leadership attributes appear to support qualification: empowerment, trust, engagement, communication, clarification, governance and consistency, and fit for purpose (Albrecht and Andreetta, 2011; Antony *et al.*, 2007; Kent, 2006; Marinelli-Poole *et al.*, 2011; Steed, 2012; Taher *et al.*, 2016; Willis *et al.*, 2016).

Qualification rests on empowering leadership and mutual trust. Empowering leaders actively encourage and enable followers to become leaders themselves. Empowering leadership behaviors include leading by example, sharing information, coaching, and showing concern for employees (Albrecht and Andreetta, 2011); notably, these behaviors mirror leadership behaviors frequently discussed in the lean literature (Aij, Visse and Widdershoven, 2015; Al-Balushi *et al.*, 2014; Drotz and Poksinska, 2014). Al Balushi *et al.* (2014) found that training can encourage employees to become involved in lean thinking. Trust is connected to an increase in employees’ responsibilities and leads to decreased involvement of the leader (Kent, 2006). Sub-attributes of trust identified were ownership and credit to owners. The leader earns employees’ trust by demonstrating expertise, humility, interest, and fairness (Caldwell, 2014). Conversely, the effective leader trusts the well-qualified workforce, allowing the leader to focus on strategy and direction rather than micromanagement (Kent, 2006).

Engagement occurs when employees are guided by empowering leadership (Albrecht and Andreetta, 2011; Drotz and Poksinska, 2014). Empowering leadership leads to increased psychological empowerment and engagement of employees, which, in turn, creates greater commitment and lower turnover intention (Albrecht and Andreetta, 2011). Willis *et al.* (2016) observed that staff engagement levels depended largely upon leaders’ ability to make people feel heard; high engagement was associated with positive changes in processes.

Engagement emerged as a critical component of qualification and as a product of empowerment (Albrecht and Andreetta, 2011; Antony *et al.*, 2007; Willis *et al.*, 2016). Leaders promote engagement when they support and encourage workers, enhancing the flow of dialog through all levels of the organization (Antony *et al.*, 2007; Willis *et al.*, 2016). Likewise, when leaders are engaged, employees feel more empowered and are more likely to communicate with those in power. Willis *et al.* (2016) observed that engagement creates the opportunity for feedback on small changes, to understand how employees implement changes, and to identify resistance among employees.

Qualification of employees includes long-term development and continuous learning. Taher *et al.* (2016) suggest that development of key competencies in top management is necessary but must be complemented by a “bottom-up” approach that focuses on competency development in front-line staff. Kent (2006) describes two processes that support qualification: empowering the “we” and communicating for meaning. To empower the “we,” leaders give ownership of projects to employees, thus building mutual trust between the leader and employees. The leader should give credit to the project “owners,” thus encouraging employees to take responsibility and strive for positive outcomes. Leaders need to clarify outcomes and their connection to the vision, direction, guidance, and sense of purpose of the organization (Antony *et al.*, 2007; Berg and Black, 2014; Marinelli-Poole *et al.*, 2011); they also need to identify who is responsible and accountable for meeting performance measures. The leader should give employees increased responsibilities and authority through two-way communication, teamwork, and meetings (Drotz and Poksinska, 2014). Employees should have responsibility, otherwise there would not be a sense of ownership and performance of the task or the effectiveness of the performance (Drotz and Poksinska, 2014; Millward and Bryan, 2005).

Communication is another cornerstone of qualification. Effective leaders are supportive in their communication, thus increasing employee engagement (Antony *et al.*, 2007). They are encouraging, which promotes the flow of dialog and empowers employees (Willis *et al.*, 2016). Successful leaders communicate information well and listen carefully, thus building trust and gaining more information. They are able to communicate not only facts and data, but convey ideas, implications, and deep understanding of a subject, a skill defined as communicating for meaning (Kent, 2006).

The attribute clarification includes vision, direction, and guidance (Steed, 2012). The three sub-attributes enable employees to be responsible and held accountable for their performance or work.

Additional competencies include governance and consistency, fit for purpose, and innovation (Marinelli-Poole *et al.*, 2011). Governance and consistency maintain connection to the mission, vision, values, and goals of the organization. In addition, the leader should be able to recognize capabilities and competencies of employees, allowing the leader to identify development opportunities for employees and ensuring fit for purpose. Leaders should identify and promote talent among employees (Marinelli-Poole *et al.*, 2011). When all employees are competent and will not put others at risk, confidence grows. Consistency provides the means to assess competencies across multiple disciplines in complex operating environments (Marinelli-Poole *et al.*, 2011). This finding mirrors Liker and Meier’s (2007) observation that people are the most essential part of the lean system, which rests upon all employees’ ability and willingness to identify and solve problems. When creativity and talent is not fostered in employees, *muda* (waste) occurs (Liker and Meier, 2007).

#### Gemba

The principle of *gemba* is central to lean leadership, emphasizing the core concept that leaders must be involved with the people who do the work that adds value (Aij, Visse and Widdershoven, 2015; Al-Balushi *et al.*, 2014; Toussaint and Berry, 2013; Womack and Jones, 2003). In an ethnographic study of contemporary lean leadership in health care, Aij, Visse and Widdershoven (2015) describe “going to *gemba*” as an essential component in lean leadership and a critical problem-solving tool. While the action of the *gemba* walk is straightforward – the leader goes to the place where work happens – certain attitudes and attributes of the leader are key to successful *gemba* walks. Effective leaders approach *gemba* walks with care, recognition, engagement, a focus on leader-employee relationships, communication, and fairness (Aij, Visse and Widdershoven, 2015; Braithwaite *et al.*, 2007; Glavin and Chilingerian, 2010; Kent, 2006; Steed, 2012). In addition, *gemba* walks should be done publicly, and time must be set aside for the visits to the front line. These findings echo Toussaint and Berry’s (2013, p. 8) suggestion that lean leaders set aside a two-hour “meeting-free zone” each day for *gemba* walks, during which they should respectfully seek input from front-line workers through Socratic questioning (Toussaint, 2015).

The effective lean leader demonstrates care and recognition during *gemba* walks by recognizing the value of employees’ work, being encouraging, and giving emotional rewards (Antony *et al.*, 2007; Kent, 2006). The leader should show that she cares about and recognizes the person and the work delivered by making regular, highly visible visits, so the leader can answer questions from all employees and respond to results on visual metrics boards (Aij, Visse and Widdershoven, 2015; Steed, 2012). Sub-attributes of recognition are encouragement, emotional rewards – presence, visibility, and attention – and acknowledgment. The leader should pay attention to the work and performance of employees and acknowledge it. Toussaint and Berry (2013) observe that lean “turns leadership upside down,” (p. 79) requiring senior management to trust and support front-line workers to innovate and solve problem. When a leader does not value or acknowledge employees, the employees will be less responsive to the organization and the needs of the client or patient (Kent, 2006; Steed, 2012). Leaders who demonstrate recognition through the sub-attributes demonstrate that they value employees’ work, see the work delivered, and see employee performance.

The attribute engagement enables trust through the ability of a leader to be honest, benevolent, and express well-intentioned behavior (Braithwaite *et al.*, 2007). When the employees of the leader are clinicians and doctors, engagement through trusted leadership has a positive impact on clinical and organizational performance (Hewison *et al.*, 2013). The leader must gain employees’ trust by being honest and benevolent, and displaying well-intentioned behavior (Aij, Visse and Widdershoven, 2015; Braithwaite *et al.*, 2007; Kent, 2006). In the leader-employee relationship, trust evolves from reliance and confidence in the integrity of each other. Communication is key for the development of trust, as it involves the exchange of verbal and non-verbal information (Braithwaite *et al.*, 2007). The ability to read non-verbal cues and expressions is important to understanding someone’s intentions. Trust is enabled through the reliance and confidence in the leader-employee relationship. These attributes correspond with the *gemba* principle of working with a small leader-to-employee ratio (Dombrowski and Mielke, 2014).

A fair process is essential to the leader’s ability to generate and maintain trust (Glavin and Chilingerian, 2010). A fair process consists of engagement, explanation, and clarity of expectations. Engagement involves employees in decisions, explanation makes employees understand why decisions are made, and clarity of expectations provides employees with a set of rules that apply to the new standards. The leader must “talk the talk and walk the walk” (Glavin and Chilingerian, 2010). Unfortunately, this process is not used frequently, as many leaders fear it will lead to loss of power and control; and do not think that employees will prioritize decisions that are best for the organization, instead of themselves (Glavin and Chilingerian, 2010).

#### Hoshin kanri

The lean principle of *hoshin kanri* calls for a focus on the customer and alignment of goals on all levels. Lean leaders in health care must keep both elements at the forefront as they work to improve patient care and processes across the organization. Toussaint and Berry (2013) use the term “unity of purpose” to convey the coordination, alignment, and prioritization required for the organization to achieve *hoshin kanri*. Four key leadership attributes emerged from this research that support the principle of *hoshin kanri*: define and provide value; demonstrate principles of lean; communication; and role adaptation (Drotz and Poksinska, 2014; Al-Balushi *et al.*, 2014; Pronovost and Marsteller, 2014; Angood and Shannon, 2014; Marinelli-Poole *et al.*, 2011; Steed, 2012).

In successful lean initiatives, value is defined and provided across the hierarchy of the health-care organization (Al-Balushi *et al.*, 2014; Antony *et al.*, 2007). There are many customers in health-care organizations, including patients and their relatives, caregivers, commissioners, taxpayers, insurance companies, and others (Al-Balushi *et al.*, 2014). The lean leader must define value for each group (Al-Balushi *et al.*, 2014; Pronovost and Marsteller, 2014). The leader who defines and provides value offers the sub-attribute of autonomy to employees, which, in turn, reduces resistance to change (Drotz and Poksinska, 2014).

Lean leaders who demonstrate their knowledge of lean principles are more effective at breaking down hierarchies and aligning goals across the organization (Aij, Visse and Widdershoven, 2015; Al-Balushi *et al.*, 2014; Taher *et al.*, 2016). Because health-care organizations tend to by hierarchically structured, strong support for lean from the leadership team is essential (Al-Balushi *et al.*, 2014). The lean leader should demonstrate the principles of lean through the sub-attributes of support, commitment, and understanding (Al-Balushi *et al.*, 2014). By demonstrating the principles, the leader enables staff to enact them as well.

Clear communication was identified as an important competency of the health-care leader and, in the lean literature, essential to *hoshin kanri* (Angood and Shannon, 2014; Pronovost and Marsteller, 2014; Marinelli-Poole *et al.*, 2011). Leaders should regularly communicate with employees across all levels of the organization to ensure that information is disseminated and to learn about employees’ experiences, problems, and suggestions. Communication is enhanced by the leader who remains in contact, is able to clarify, and conveys information concisely and clearly. Communication is used by health-care leaders to facilitate contact, improve performance, hold employees accountable, and creates and maintain relationships. The leader should be able to communicate with internal and external customers, creating and maintaining relationships with both individuals and groups. Moreover, the leader must adapt to special roles, such as champion and as cheerleader, to make sure that messages will be clearly communicated and formulated into the form of “what, why, and how” (Steed, 2012).

### Complexity of attributes

Attributes identified in this research reveal a complex web of influence on leadership behaviors. Although we have correlated each attribute with a specific principle, several attributes appear across principles. In addition, while certain attributes may appear similar, their effects can differ greatly.

For instance, “responsible autonomy,” a sub-attribute of the attribute “autonomy” in improvement culture, has the effect of giving employees authority over their tasks and enabling them to participate fully in the organization (Drotz and Poksinska, 2014). Responsible autonomy resembles the sub-attribute “autonomy” of the attribute “define and provide value” in *hoshin kanri*. However, in the context of *hoshin kanri*, autonomy enables the lean leader to reduce resistance to change (Drotz and Poksinska, 2014), and in terms of the principle qualification, responsibility is an effect of the attribute “trust” (Kent, 2006).

“Trust,” likewise, spans multiple principles. In the context of *gemba,* trust results when engaged leaders actively build leader-employee relationships by interacting with employees at the front line (Aij, Visse and Widdershoven, 2015; Braithwaite *et al.*, 2007; Toussaint and Berry, 2013). The attribute “honesty” is essential to building trust. Likewise, honesty is a primary attribute of improvement culture and enables the leader to acknowledge and learn from errors (Caldwell, 2014; Steed, 2012). In terms of qualification, trust is an attribute that supports employee development by allowing employees to assume responsibility and leaders to step back.

“Communication” is an attribute common to three principles: qualification, *gemba*, and *hoshin kanri*. In qualification, communication, which is both verbal and non-verbal, is the key to involving employees and developing trust at the front line (Braithwaite *et al.*, 2007). In the context of *gemba*, the leader uses communication to connect with the employee and assess failures and errors of processes. And in *hoshin kanri*, communication is characterized by the sub-attributes contact, clarification, and concision and clarity (Angood and Shannon, 2014; Pronovost and Marsteller, 2014; Marinelli-Poole *et al.*, 2011). These sub-attributes allow the leader to facilitate contact, improve performance, hold employees accountable, and create and maintain relationships. Communication enables the leader to improve customer value and convey information and ideas across hierarchical systems of health care.

“Support” spans the principles of self-development, qualification, and *hoshin kanri*. In self-development, the attribute “visualizing greatness” is characterized by sub-attribute “support with enthusiasm” and “support positively.” The effect of support is to “encourage inspiration.” In qualification, the attribute “communication” is enabled by the sub-attribute “support” and enables the leader to create engagement, flows of dialogues, and empowerment from employees. *Hoshin kanri*, likewise, includes the sub-attribute “support” as an element of the attribute “demonstrate the principles of lean,” with the effect of eliciting the “required behavior of the staff in the hierarchical structure.” Thus, the leader who is skilled at providing support can better achieve different effects on each principle. It is also notable that support is a sub-attribute of an attribute for each principle.

Our findings replicate and expand upon Jim Collins’ (2001) cross-industry findings that the most transformative leaders possess a combination of the attributes “humility” and “will” (i.e. determination). We have been able to break these down into their component parts and identify their specific links to lean leadership principles articulated by Dombrowski and Mielke (2013). This finding suggests that even in complex, hierarchical health-care settings, Collins’ observations still apply.

The resemblance of attributes across principles is a new finding, which might be explained by Dombrowski’s model (2013), in which the principles are connected through the concept of team. The concept of team is necessary in a lean environment, and the lean leader cooperates with, corrects, and directs each team so it aligns with other teams in the organization.

All examples of attributes and sub-attributes that resemble attributes in different principles have a different effect on the organization or employees. The desired effect of the attributes needs to be identified by the lean leader. The overview in the annex of the attributes per principle should help the lean leader to distinguish the attribute needed to match the desired effect.

### Implications

Through this research, we have identified specific behaviors and attributes of leaders in health-care settings and described their association with the five core principles of lean leadership described by Dombrowski and Mielke (2013). Our results offer health-care leaders and organizations the means to consciously choose and learn behaviors and attributes that contribute to successful lean implementation. Several attributes were found to span one or more principles, suggesting that the leader who displays these attributes can simultaneously influence several lean principles at once.

This analysis of lean leadership attributes offers opportunities for further study and application. A strength is the overview of necessary lean leadership attributes per principle in the annex. The overview enables the viewer or lean leader to make use of the lean leadership attributes combined with the desired effect per lean principle. This kind of overview of lean leadership attributes and its applicability cannot be found in the literature currently.

### Limitations

There are several limitations to this study. Selection criteria were focused on lean in health care, limiting the understanding of leadership attributes to one sector. Most of the articles reviewed are about the implementation of lean or other leadership models and do not focus primarily on the attributes of effective leaders. As the research method was specifically focused on the combination of lean and leadership in health care, most data were found in fragments of the articles. Few articles were fully dedicated to the topic of lean leadership attributes.

The majority of leadership articles in this review were based on subjective assessments of behaviors. Little empirical evidence was found to explicitly link leadership attributes with organizational outcomes. The paucity of work on attributes of lean leaders in health care suggests that more work should to be done in this area. Additional data linking statistical analyses, including ranking of leadership attributes and effects are needed. In addition, articles selected for review were sifted and analyzed by one person, increasing the risk of inclusion bias.

The lean leadership attributes found are specifically related to the health-care sector and may not be applicable to other industries. Existing knowledge and information about leadership attributes in other industries should be compared with the outcomes of this research.

Much further work needs to be done, including empirical research into the behaviors and attributes of successful lean leaders in health care. In addition, more research is needed to specify ways in which the leadership attributes may affect lean success differently in various health-care settings such as hospitals, clinics, and public health systems.

## Conclusion

This work contributes to the development of a new framework for describing leadership attributes within lean management of health care, founded on the lean leadership principles described by Dombrowski and Mielke (2013). Lean leadership attributes provide a guide for the lean leader and enable the leader to adjust behavior to achieve the desired effects on employees and organizational processes. Several attributes identified here resemble each other, demonstrating the complex relationship between leadership attributes and principles. This summary of attributes can provide a model for health-care leaders to apply lean in their organizations.

## Figures and Tables

**Table I:** Inclusion and exclusion criteria

Inclusion criteria	Exclusion criteria
Articles in the period of 2000-2016	Articles written before the year 2000
Articles that connected lean managementto health care	Articles that did not cover lean management in health care
Articles that were connected to leadership	Articles that were not connected to leadership
Articles that were connected to leadership attributes	Articles that were not connected to leadership attributes
English articles	Other languages than English

**Table II:** Improvement culture attributes

Attributes	Sub-attributes	Effect	Author
Task identity		Finalize problems	Drotz and Poksinska (2014)
Feedback		Give to the employees Allocation of time Resources Creation of supporting structures	Drotz and Poksinska (2014)
Autonomy	Choice responsible	Standardization Authority, participation, etc.	Drotz and Poksinska (2014)
Believe in improvement	Understand lean principles	Improve culture	Crump (2008)
Honesty		Make errors Learn from errors	Caldwell (2014), Steed (2012)

**Table III:** Self-development attributes

Attributes	Sub-attributes	Effect	Author
Show interest Facilitate resources		Enhance staff behavior for lean implementation	Al-Balushi *et al.* (2014)
Emotional intelligence		Increase self-awareness	Hopkins *et al.* (2011)
Visualizing greatness	Support with enthusiasm Support positively Communicate vision	Encourage inspiration	Kent (2006)
Aware of its status	Behavior Thinking Feelings	Awareness and maintenance of its presentation	Kent (2006)
Skills	Motivate Inspire Stimulate Facilitate	Be effective	Millward and Bryan (2005)

**Table IV:** Qualification attributes

Attributes	Sub-attributes	Effect	Author
Empowerment		Commitment and motivation of employees	Albrecht and Andreetta (2011)
Trust	Ownership Credit to owners	Responsibility Be less involved	Kent (2006)
Engagement		Commitment and motivation of employees Feedback Understand Identify resistance	Willis *et al.* (2016)
Communication	Supportive Encouraging Listen	Engagement Flows of dialogs Empowerment Gain information Truism behavior	Antony, *et al.* 2007), Willis *et al.* (2016)
Clarification	Vision Direction Guidance	Responsibility and Accountability	Steed (2012)
Governance and consistency		Guide to right direction	Marinelli-Poole *et al.* (2011)
Fit for purpose		Competent employees Less risk Induce competence	Marinelli-Poole *et al.* (2011)

**Table V:** *Gemba* attributes

Attributes	Sub-attributes	Effect	Author
Care		Value for work Importance of work	Kent (2006)
Recognition	Encouraging and Emotional rewards Presence Visible Pay attention Acknowledge	Value work See work delivered See performance	Aij, Visse and Widdershoven (2015), Kent (2006), Steed (2012)
Engagement	Honesty Benevolent well-intentioned behavior	Trust	Aij, Visse and Widdershoven (2015), Braithwaite *et al.* (2007)
Leader-employee relationship	Reliance Confidence	Trust Integrity between leader and employee	Braithwaite *et al.* (2007)
Communication	Verbal Non-verbal	Development of trust	Braithwaite *et al.* (2007)
Fairness	Engagement Explanation Expectation clarity	Involvement Understanding Rules and standard	Glavin and Chilingerian (2010)

**Table VI:** *Hoshin kanri* attributes

Attributes	Sub-attributes	Effect	Author
Define and provide value	Autonomy	Reduce resistance to change	Drotz and Poksinska (2014)
Demonstrate principles of lean	Support Commitment Understanding	Required behavior of staff in the hierarchical structure	Al-Balushi *et al.* (2014)
Communication	Contact Clarify Concise manner Clear manner	Facilitate contact Improve performances Accountability of employees Create relationships Maintain relationships	Pronovost and Marsteller (2014), Angood and Shannon (2014), Marinelli-Poole *et al.* (2011)
Role adaptation	Champion Cheerleader	Improve communication and formulation	Steed (2012)
